# Supplementary material for: Pressure-Induced Chemical Bonding Effects on Lattice and Magnetic Instabilities in Antiferromagnetic Insulating CaMn2Sb2
Source: ACS Mater Au. 2026 Mar 19;6(3):633–41. doi: 10.1021/acsmaterialsau.6c00021 (PMC13177399; doi:10.1021/acsmaterialsau.6c00021)

Supplementary Information

Pressure-Induced Chemical Bonding Effects on Lattice and Magnetic Instabilities in Antiferromagnetic Insulating $CaMn_2Sb_2$

Matt Boswell[1], Antonio M. dos Santos[2], Mingyu Xu[1], Madalynn Marshall[3], Su-Yang Xu[4], Weiwei Xie[1*]

1. Department of Chemistry, Michigan State University, East Lansing, MI 48824 USA
2. Neutron Scattering Division, Oak Ridge National Laboratory, Oak Ridge, TN 37831 USA
3. Department of Chemistry and Biochemistry, Kennesaw State University, Kennesaw, GA 30144 USA
4. Department of Chemistry and Chemical Biology, Harvard University, Cambridge, MA 02138, USA

Corresponding Author: Weiwei Xie (xieweiwe@msu.edu)

Table of Contents

Table S1. Refinement information of $CaMn_2Sb_2$ at various pressures……………………..……S2

Table S2. Atomic information of $CaMn_2Sb_2$ at various pressures…………………………..……S3

Figure S1. Resistivity measurement on synthesized crystal of $CaMn_2Sb_2$….……………………..S4

Figure S2. Lattice parameters as a function of pressure …………………………………………S5

Figure S3. Lowest occupied molecular orbitals (LOMOs) of Mn@Sb4 layers………………….S6

Figure S4. Atomic distance evolution in Mn-Sb…………………………………………………S7

Table S1. The crystal structure and refinement of $CaMn_2Sb_2$ at room temperature and various pressures. Values in parentheses are estimated standard deviation from refinement.

**Pressure**	**0 GPa**	**4.5 GPa**	**5.9 GPa**
Space Group	P-3*m*1	P-3*m*1	$P2_1/m$
Lattice Parameters (Å)	*a* = 4.5359(1)<br>*c* = 7.4930(3)	*a* = 4.4097(3)<br>*c* = 7.278(1)	*a* = 7.2708(15)<br>*b* = 4.1436(8)<br>*c* = 7.3702(15)<br>β = 93.88(3)°
Volume (Å^3)	133.51(1)	122.57(3)	221.54(8)
Absorption coefficient	15.407 mm^{-1}	16.783 mm^{-1}	9.285 mm^{-1}
F (000)	172	172	172
θ range (°)	2.72 to 41.14	5.34 to 36.26	5.55 to 36.23°
Reflections collected	8576	3285	1394
Independent reflections	382	152	293
Refinement method	F^2	F^2	F^2
Data/restraints/parameters	382/0/9	152/0/16	293/0/17
Final *R* indices	$R_{1(I>2\sigma(I))}$ = 0.0238;<br>$wR_{2(I>2\sigma(I))}$= 0.0558<br>$R_{1(all)}$ = 0.0257;<br>$wR_{2(all)}$ = 0.0562	$R_{1(I>2\sigma(I))}$ = 0.0651;<br>$wR_{2(I>2\sigma(I))}$= 0.1585<br>$R_{1(all)}$ = 0.1377;<br>$wR_{2(all)}$ = 0.2416	$R_{1(I>2\sigma(I))}$ = 0.4012;<br>$wR_{2(I>2\sigma(I))}$= 0.6405<br>$R_{1(all)}$ = 0.6129;<br>$wR_{2(all)}$ = 0.7301
Largest diff. peak &hole	+5.245 e^-/Å^3<br>-0.719 e^-/Å^3	+7.869 e^-/Å^3<br>-10.165 e^-/Å^3	+1.861 e^-/Å^3<br>-3.173 e^-/Å^3
R.M.S. deviation	0.318 e^-/Å^3	0.947 e^-/Å^3	1.028 e^-/Å^3
Goodness-of-fit on F^2	1.087	1.264	2.529

Table S2. Atomic coordinates and equivalent isotropic atomic displacement parameters (Å^2) of $CaMn_2Sb_2$ at room temperature and various pressures. (U_{eq} is defined as one-third of the trace of the orthogonalized U_{ij} tensor.)

0 GPa

Atoms	Wyck.	*x*	*y*	*z*	Occ.	U_{eq}
**Ca**	1*a*	0	0	0	1	0.015(1)
**Mn**	2*d*	1/3	2/3	0.6230(1)	1	0.015(1)
**Sb**	2*d*	1/3	2/3	0.2514(1)	1	0.013(1)

4.5 GPa

Atoms	Wyck.	*x*	*y*	*z*	Occ.	U_{eq}
**Ca**	1*a*	0	0	0	1	0.035(6)
**Mn1**	2*d*	1/3	2/3	0.4027(2)	0.813	0.001(1)
**Mn2**	2*d*	1/3	2/3	0.280(5)	0.132	-0.109(1)
**Mn3**	2*d*	1/3	2/3	0.078(7)	0.067	-0.103(1)
**Sb**	2*d*	1/3	2/3	0.7512(8)	1	0.040(2)

5.9 GPa

Atoms	Wyck.	*x*	*y*	*z*	Occ.	U_{iso}
**Ca**	2*e*	0.028(1)	¼	0.710(1)	1	0.09(1)
**Mn1**	2*e*	0.535(1)	¼	0.797(1)	1	0.20(1)
**Mn2**	2*e*	0.420(1)	¼	0.442(1)	1	0.14(1)
**Sb1**	2*e*	0.273(1)	¼	0.074(1)	1	0.16(1)
**Sb2**	2*e*	0.813(1)	¼	0.364(1)	1	0.17(1)

Figure S1. Resistivity measurement on synthesized crystal of $CaMn_2Sb_2$

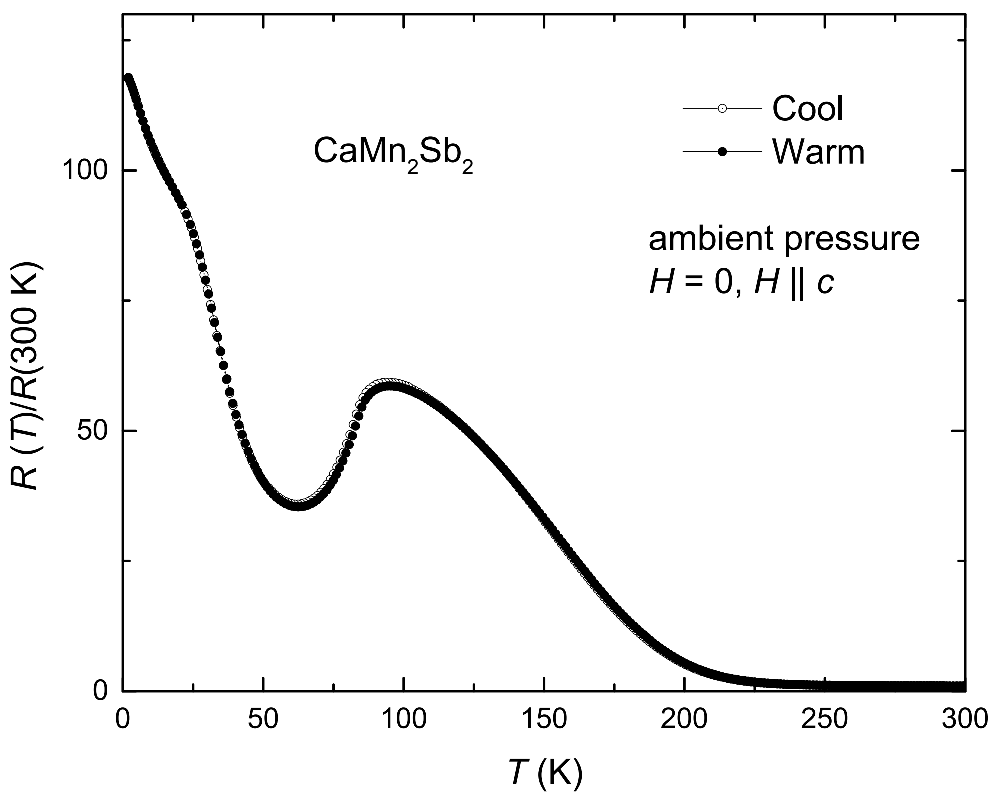

Figure S2. Normalized lattice parameters to initial lattice values at 0 GPa.

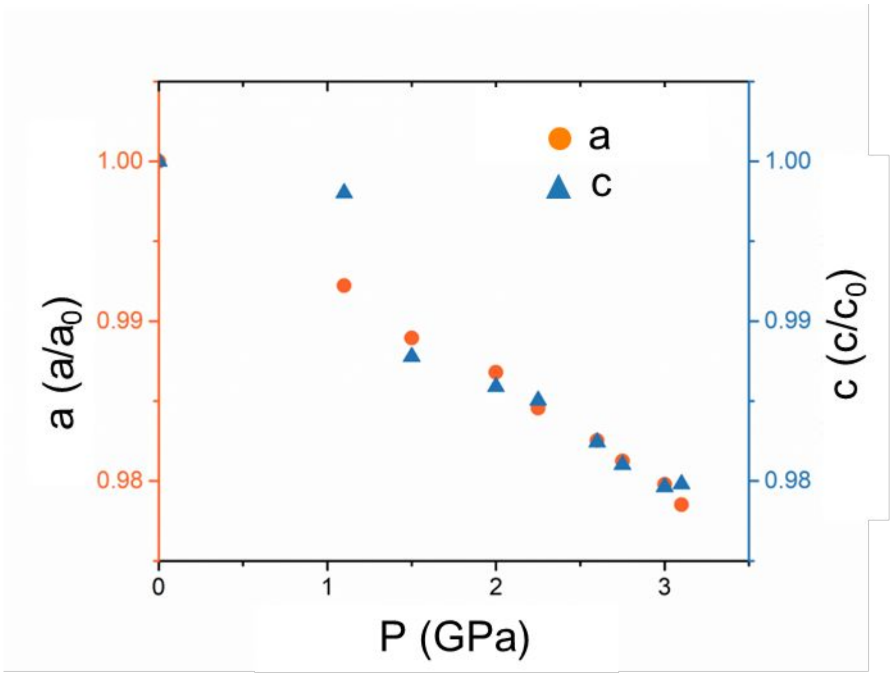

Figure S3. Lowest occupied molecular orbitals (LOMOs) of Mn@Sb4 layers in ***(a)*** the tetragonal phase and ***(b)*** the trigonal phase.

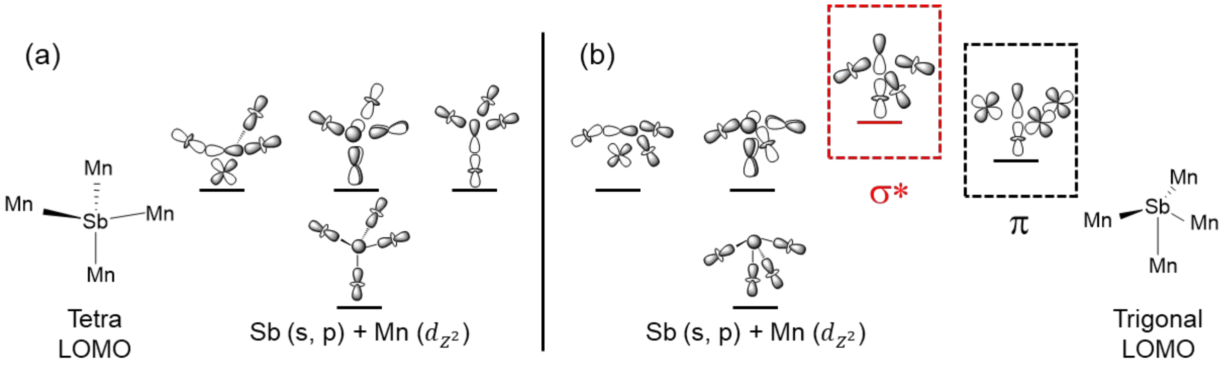

Figure S4. Atomic distance evolution in Mn-Sb

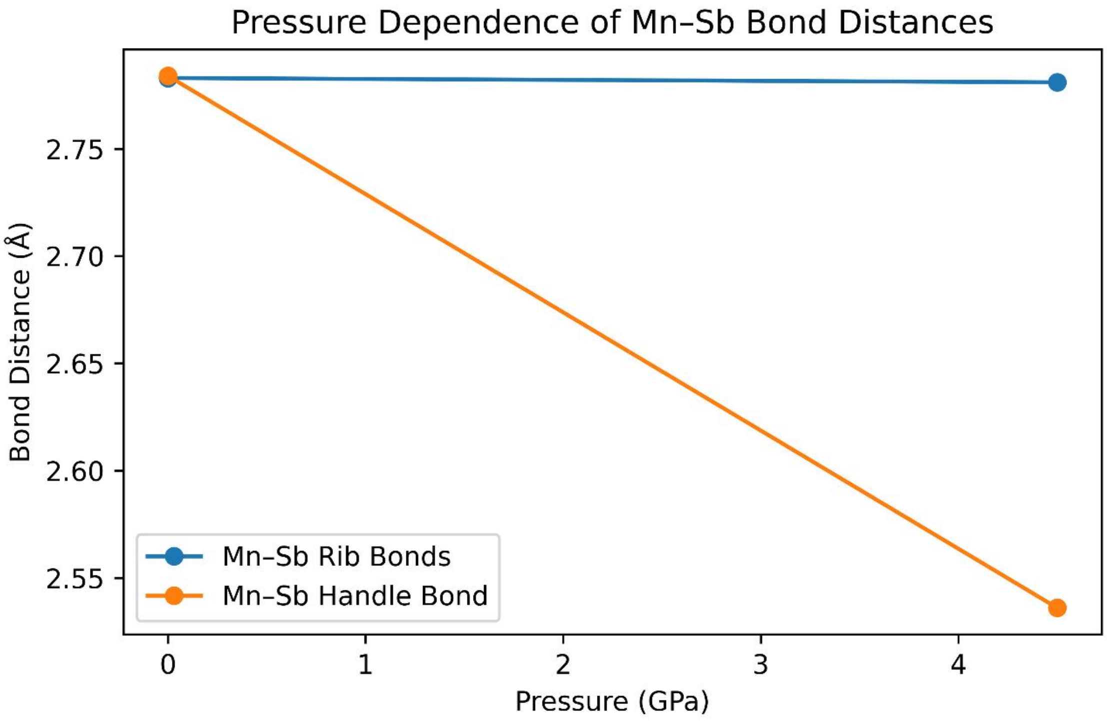

Supplement: Supplementary file 1 [file mg6c00021_si_001.pdf]
